# A rapid equity focused health impact assessment of a policy implementation plan: An Australian case study and impact evaluation

**DOI:** 10.1186/1475-9276-10-6

**Published:** 2011-01-30

**Authors:** Ben F Harris-Roxas, Patrick J Harris, Elizabeth Harris, Lynn A Kemp

**Affiliations:** 1 Centre for Health Equity Training, Research and Evaluation (CHETRE), part of the UNSW Research Centre for Primary Health Care and Equity, School of Public Health and Community Medicine, University of New South Wales, Sydney, Australia

## Abstract

**Background:**

Equity focused health impact assessments (EFHIAs), or health equity impact assessments, are being increasingly promoted internationally as a mechanism for enhancing the consideration of health equity in the development of policies, programs and projects. Despite this there are relatively few examples of examples of completed EFHIAs available. This paper presents a case study of a rapid EFHIA that was conducted in Australia on a health promotion policy implementation plan. It briefly describes the process and findings of the EFHIA and evaluates the impact on decision-making and implementation.

**Methods:**

The rapid EFHIA was undertaken in four days, drawing on an expert panel and limited review of the literature. A process evaluation was undertaken by email one month after the EFHIA was completed. An impact evaluation was undertaken two years later based on five semi-structured interviews with members of the EFHIA working group and policy officers and managers responsible for implementing the plan. A cost estimation was conducted by the EFHIA working group.

**Findings:**

The EFHIA made both general and specific recommendations about how the health equity impacts of the policy implementation plan could be improved. The impact evaluation identified changes to development and implementation that occurred as a result of the EFHIA, though there was disagreement about the extent to which changes could be attributed solely to the EFHIA. Those responsible considered the recommendations of the EFHIA in the next versions of their ABHI implementation plans. Factors that influenced the impact of the EFHIA included consolidating understandings of equity, enabling discussion of alternatives, and differing understandings of the purpose of the EFHIA. The EFHIA cost US$4,036 to undertake.

**Conclusions:**

This EFHIA was conducted in a short timeframe using relatively few resources. It had some reported impacts on the development of the implementation plan and enhanced overall consideration of health equity. This case highlights some of the factors and preconditions that may maximise the impact of future EFHIAs on decision-making and implementation.

## Background

There is now strong policy support internationally for governments and institutions to routinely assess the health impacts of major policies, plans, programs and projects on health to address health inequalities [1-9]. Over the past 15 years health impact assessment (HIA) has been promoted as a mechanism through which such assessment can be achieved in a structured and transparent way [10-12]. There are now many countries that have extensive experience in the ways in which HIA can add value to policy and planning decision-making processes, with activity occurring in Europe, South-East Asia, Australia, New Zealand and the USA [7,13-26].

HIA enables the systematic consideration of health inequalities early on within the development of policies and other initiatives prior to their implementation [10,27]. In doing so, HIA becomes a practical policy intervention that can shift the rhetoric of healthy public policy into action [28]. However despite this promise, the experience of the last decade demonstrates that HIA has been difficult to institutionalise within policy development cycles [29]. Further, despite equity being a conceptual driver for HIA's use [27,30], evidence and commentary suggests this has had limited translation into practice [10,31-35].

The difficulty in using HIA at the policy level has been linked to concerns about HIA fitting within the (often short) timeframes associated with the development, announcement and implementation of policies [35-38]. This concern is in part linked to one aspect of the historical development of HIA as a field, as a part of regulatory project impact assessment [39-41], which conventionally follows a more structured planning process than policy. That the development and implementation of policy is less linear and less clear than project development poses a challenge to the step-wise process of HIA [42-44].

Health equity may be discussed in little detail within HIAs. This may be due to a number of factors. Firstly, there may be few opportunities to describe and discuss what potential health impacts are considered unfair. Secondly, there may be a lack of clarity about which differential health impact should be examined. In other words: how do we know who it is unfair for? Thirdly, it maybe be unclear what changes could remedy this unfairness or injustice [16,31-33,45-47].

These challenges can be compounded by a lack of existing evidence about which groups will be disproportionately affected by the type of proposal being assessed [32,33]. Explicitly considering the broader determinants of health, beyond biomedical impacts, and engaging communities within the assessment process may help to ensure equity impacts are considered. These may not equate with considering equity, nor do they automatically lead to consideration of differential impacts, fairness or whether unfair impacts could be avoided [16,31]. Examining differential impacts can add complexity to an already conceptually difficult HIA process that is usually undertaken in an interdisciplinary and intersectoral context [48].

Recognition of the need for a framework for rapidly assessing the health equity impacts of proposed policy and program proposals led to the development of rapid equity focused health impact assessment (EFHIA, see Table 1), informed by earlier work undertaken in developing a framework for EFHIA [46,47]. This paper presents rapid EFHIA as an approach and details the process and impacts of a rapid EFHIA that was undertaken over four working days on components of a complex state-wide health promotion initiative focusing on the prevention and early detection of chronic disease. It describes the context in which the EFHIA was undertaken, the methods used for the EFHIA and to evaluate the process and impacts of the EFHIA, the findings of the EFHIA and of the evaluation, and conclusions.

**Table 1:** Health Impact Assessment-Related Terminology

Term	Explanation
Health impact assessment (HIA)	HIA is "a combination of procedures, methods and tools by which a policy, program or project may be judged as to its potential effects on the health of a population, and the distribution of those effects within the population" [27].

Health equity impact assessment (HEIA)	HEIA has been suggested as a means to ensure that the potential impacts of a proposal on health equity is considered prior to implementation [4,49]. It is related to the notion of health inequalities impact assessment that was originally proposed a decade ago in the Acheson Review in the UK [12,50]. Despite these calls, specific guidance on how to conduct HEIAs has not been developed and there are ongoing debates about whether it is possible or desirable to conduct an impact assessment focused solely on health equity without considering more general health impacts [51,52].

Equity focused health impact assessment (EFHIA)	EFHIA is related to HEIA and was developed in response to concerns that (i) consideration of health equity is often limited within HIAs, often being restricted to the realm of professed values and aspirations [31], and (ii) that it was desirable to improve the methods for considering equity within HIA, rather than developing a separate form of HEIA [52]. The term was first used in the *Jakarta Declaration on Leading Health Promotion *[53] and subsequently in the *Bangkok Declaration *[54], but was operationalised with the development of the *Equity Focused Health Impact Assessment Framework *[46,47,55] in 2004. EFHIA focuses on improving the consideration of equity and differential impacts at each step of the HIA process [46,47]. A rapid EFHIA involves scoping the EFHIA so it can be conducted within a limited time frame with limited resources [56].

## Context

The NSW Department of Health *Australian Better Health Initiative (ABHI) Implementation Plan *was developed in 2006 as part of a Council of Australian Governments (COAG) reform package aimed at achieving better health for all Australians through a focus on the prevention and early detection of chronic disease [57]. The implementation plan looked at the implementation of the health promotion-related components of the ABHI in New South Wales (NSW), a state of seven million people in eastern Australia. In NSW, the prevention and early intervention initiatives and their supporting strategies needed to be developed within a short timeframe to enable resources to be allocated within the funding period identified in the COAG agreement.

The draft implementation plan was sent by the NSW Department of Health to key stakeholders for comment. The Centre for Primary Health Care and Equity at the University of New South Wales was included in this process due to its expertise in chronic disease prevention. Centre staff noted that despite equity appearing within the background to the document as a value there was little explicit focus on equity within the strategies and approached the NSW Department of Health about conducting a rapid EFHIA on the initiatives. This was agreed by those developing the initiative within the Department and they were receptive to an EFHIA being conducted provided that (i) it could be done within four working days as the final document needed final approval three days after this deadline, (ii) did not suggest new strategies but made recommendations on how existing strategies could be strengthened or modified, and (iii) did not recommend changes in funding levels. Issues related to Aboriginal health, though important in any consideration of health equity impacts in the Australian context, were excluded from consideration within the rapid EFHIA as these were being covered through a separate Aboriginal Health Impact Statement process.

## Methods

### Rapid EFHIA methods

A structured approach to screening and scoping the HIA was undertaken by the EFHIA working group using the NSW guide for HIA [56]. The core EFHIA working group was made up of three staff from CHETRE and one NSW Department of Health employee, all of whom were experienced in conducting HIAs. Additionally an expert panel was recruited to undertake the HIA assessment step. Each expert panel member agreed to attend one six hour assessment workshop (Day 2), comment of drafts and participate in two one-hour teleconferences (Days 3 and 4, see Table 2). The expert panel had nine members, seven of whom were able to attend the workshop. They included people with expertise in health equity, early intervention, health promotion, chronic disease prevention, and policy analysis.

**Table 2:** Timeline for the rapid EFHIA

Day	Day 1	Day 2	Day 3	Day 4
**Who**	EFHIA working group	EFHIA working group and expert panel	EFHIA working group and expert panel	EFHIA working group and expert panel

**HIA step **[52]	Screening and scoping	Identification and assessment of impacts (appraisal)	Negotiation and decision-making	Negotiation and decision making

**Activity**	Screening and scoping report, identification of key documents & organisation of expert panel	Appraisal workshop, drafting report	Teleconference, drafting report	Teleconference, drafting report

The purpose of the screening report was to identify the potential links between the implementation of the initiatives, health improvement and potential health inequities. The screening report determined that the initiatives had the potential to improve health but also to have differential impacts across the population.

The scoping report (included in Additional File 1) established terms of reference for the EFHIA and expert panel, clarified definitions of health and equity, determined the dimensions on which differential impacts were to be considered in the EFHIA (age, gender, place of residence, ethnicity and socioeconomic position), the process by which the EFHIA would be undertaken, and clarification of values and assumptions, especially in defining health, equity and inequity. Of particular importance was a decision to make recommendations that would positively impact on the whole of the population (mainstream approaches) as well as those that would specifically focus on particular groups (targeted approaches). Due to time constraints it was agreed that the assessment process be principally based on expert opinion, supported by a small number of reports providing data on inequity in relation to chronic disease [58,59].

At the appraisal workshop the screening and scoping papers were discussed, refined and accepted. The group then systematically worked through each of the eight strategies included in the assessment, addressing five specific questions:

1. What is the initiative trying to do?

2. Is there evidence of inequity?

3. Who may be disadvantaged by the initiative?

4. Are there likely to be unanticipated impacts?

5. What are the key recommendations for implementation?

These questions reflected aspects of the EFHIA framework [46], drew on work that had been undertaken by members of the EFHIA working group on the development of Australian National Health and Medical Research Council Guidelines [60], and are similar to other questions used in other equity audits and HIA screening tools [61-64].

To inform their decision-making, the group made an assessment of the potential size of the impact of the initiative on health, the likelihood of the impact and the groups who may be affected. The resulting EFHIA appraisal for each initiative was summarised in one page to facilitate use by the Department in the short timeframe within which they were preparing the implementation plan.

Drafts of the EFHIA report were circulated to members at the end of each day and teleconferences were held early on Days 3 and 4. A draft EFHIA report was sent to the NSW Department of Health on the morning of Day 5. The final document, which incorporated modifications based on comments received from the Department of Health, was sent on Day 7 [65].

### Evaluation methods

#### Process evaluation methods

A brief process evaluation [66,67] was undertaken through panel members being asked to reflect on the experience via email and what they perceived as the strengths and weaknesses of the process. This was supplemented by a brief discussion one month after submission of the report with the officers and managers responsible for the NSW ABHI implementation plan.

#### Impact evaluation methods

To evaluate the impact of the EFHIA on planning and implementation, five semi-structured interviews were conducted two years after the EFHIA was completed. The attributes of these interviews are described in Table 3 using the CORE-Q consolidated criteria for reporting qualitative research [68]. The interviewees included policy officers and managers responsible for developing and overseeing the health promotion components of the *NSW Department of Health ABHIA Implementation Plan *and members of the EFHIA working group.

Each interviewee was asked the following questions:

1. Tell me the story of the New South Wales Australian Better Health Initiative equity focused health impact assessment. (Prompt: And then what happened?)

2. What changed as a result of doing the equity focused HIA?

3. Was the equity focused HIA a success?

4. What is required for an equity focused HIA to be successful?

Interviews were audio recorded and then transcribed.

The interviews were analysed qualitatively using a modified version of the analytic method developed by Colaizzi [69,70]. The major emergent themes from this analysis are detailed in the findings section.

**Table 3:** CORE-Q Consolidated Criteria for Reporting Qualitative Research [68]

No	Item	Description
**Domain 1: Research Team and Reflexivity**		

	**Personal Characteristics**	

1	Interviewer/facilitator	Ben Harris-Roxas

2	Credentials	Master of Policy and Applied Social Research, currently enrolled in a PhD

3	Occupation	Research Fellow, University of New South Wales

4	Gender	Male

5	Experience and Training	Has undertaken several qualitative studies, trained in interviewing, qualitative analysis and using NVivo [92]

	**Relationship with participants**	

6	Relationship established	A relationship existed with all interviewees prior to the interviews

7	Participant knowledge of the interviewer	Knew the researcher has worked on HIA and health equity for several years, have had contact through other activities than the HIA described

8	Interviewer characteristics	Is doing a PhD on EFHIA

**Domain 2: Study Design**		

	**Theoretical Framework**	

9	Methodological orientation and theory	Analysis based on a modified version of Calaizzi's framework [69]

	**Participant Selection**	

10	Sampling	Purposive

11	Method of approach	Email

12	Sample size	5

13	Non participation	0

	**Setting**	

14	Setting of data collection	Participants' workplaces

15	Presence of non-participants	No

16	Description of sample	A mix of those who conducted the HIA and those who were responsible for implementing its recommendations

	**Data Collection**	

17	Interview guide	Not provided in advance, piloted on 2 interviews not included in study

18	Repeat interviews	No

19	Audio/visual recording	Audio

20	Field notes	No

21	Duration	Mean 24 minutes, Range 20 minutes (min) to 33 minutes (max)

22	Data saturation	Yes, authors decided that saturation was reached after 5 interviews

23	Transcripts returned	No

**Domain 3: Analysis and Findings**		

	**Data Analysis**	

24	Number of data coders	3

25	Description of the coding tree	No

26	Derivation of themes	Derived from the data

27	Software	NVivo [92]

28	Participant checking	No

	**Reporting**	

29	Quotations presented	Yes, each participant is numbered when quoted

30	Data and findings consistent	Yes

31	Clarity of major themes	Yes

32	Clarity of minor themes	No

#### Resource description methods

The resources involved in conducting the EFHIA were estimated by the EFHIA working group and are included to aid future cost utility studies of HIAs.

## Findings

### Rapid EFHIA recommendations

For each of the eight initiatives included in the EFHIA, a one page summary was included in the report which described in some detail the questions that guided the EFHIA. Additional File 1 includes the summaries for all the initiatives included in the EFHIA.

### Evaluation Findings

#### Process evaluation findings

The data from the process evaluation identified four factors that assisted the EFHIA. These were the support, commitment and openness of the Department of Health to having their plan assessed, the clarity of the instructions from the Department of Health (included in Additional File 1), the structured process the EFHIA followed, and the composition and experience of the expert panel coupled with the ease with which they were able to work together. Because the Centre for Health Equity Training, Research and Evaluation (CHETRE) is recognised as having expertise in this area and had worked with many of the managers on previous projects [71], there was a level of trust which facilitated the conduct of the EFHIA. The structured process made it transparent what commitment was required of the participants and the expert panel in terms of time and scope of activities to be undertaken.

Three major constraints to undertaking the EFHIA were identified. These were the timeframe required, reliance on expert opinion and a limited range of literature rather than a broader range of evidence, and the difficulty in being objective concerning negative or unanticipated consequences that individual members of the EFHIA working group or expert panel strongly supported.

For policy officers and managers the EFHIA provided an opportunity for reflection on how issues of equity had been addressed in the draft implementation plan and how these and other issues could be improved. For example what balance was needed between innovation, which often had high political and professional appeal, and expanding and sustaining existing programs for which there was evidence of effectiveness. In other words, managers had to decide if they should inject more funding and support into a new suit of programs and to neglect existing programs.

#### Impact evaluation findings

The interviews identified a number of direct and indirect changes to the NSW ABHI implementation plan that occurred as a result of the EFHIA. Though there was a high degree of concordance about the process of the EFHIA (how it was conducted, who was involved, what the major events were, etc.) there was disagreement between the interviewees about the extent of change that occurred as a result of the rapid EFHIA. This disagreement was not fundamental: all those interviewed felt that the EFHIA had some impact on further planning and decision-making. Rather the disagreement was about the extent of change that could be attributed to the EFHIA. This ranged from a very small amount of influence according to some of those interviewed, to what was regarded as a moderate amount of influence by others.

The impact evaluation identified five major themes in relation to the impact of the rapid EFHIA:

##### 1. Changing implementation planning

There were a number of changes to the NSW ABHI implementation plan that were attributed to the EFHIA. The most obvious of these were that the managers responsible for the development of aspects of the plan were asked to re-draft their sections to take the EFHIA into account.

"What we did after we got the equity focused HIA, we gave it to all the managers, and then for each one of their little, sort of, almost section in the plan, we said 'we want you to write a proper plan about how you're going to do it, and we want in your plan, to specifically say how you're going to address the recommendations of this.'

Interviewee 1

Other changes were attributed to the EFIA, though there was greater disagreement between the EFHIA working group and the Department of Health staff about the nature, extent and reasons for the changes. An example of this were the recommended changes to the proposed resource allocation split between urban and rural Area Health Services for a specific activity within the implementation plan, to favour more resources going to rural health services.

I think it actually did have at least one impact that I know of, which was that we had identified that not enough money was being invested in rural areas, although the resources were going to be allocated, the rural and the urban areas were going to get the same resources. And so I understood that... the problem is, for some of the rural areas, what they would have been getting wasn't enough to actually employ someone, so sixty thousand in the thick of an urban area's quite a lot, but in a rural area, it actually doesn't give you capacity. So I understood that what happened was each of the rural area health services was given a larger amount of money than the urban areas, and that they then wrote up their proposals... So it did have that impact.

Interviewee 2

Several interviewees identified this as a change attributable to the EFHIA but others discounted it, as the measure was not implemented in the form originally outlined in the draft implementation plan due to reallocation of funding. This mirrors some of the difficulties that have been found in other HIA impact evaluations in trying to attribute changes solely to a specific HIA [72-74].

##### 2. Consolidating understandings of equity

There was a broad agreement between the interviewees that the rapid EFHIA had brought the potential health equity impacts to the fore of the development of the implementation plan and that this was unlikely to have been as clearly addressed without the EFHIA. The interviewees from the Department of Health regarded this focus on health equity issues as a consolidation and focusing of existing knowledge, rather than being transformative or revelatory in nature - it was regarded as possibly under-considered information rather than unknown.

It probably provided a useful tool to make sure that people considered equity issues. Had we not had support for those type of [equity] issues being considered up the line, it probably would have been used as an internal advocacy tool...

...I'm not convinced that [the EFHIA] made people do things differently, because I think that they probably, should've, would've, hopefully would've, done those things anyway. It was nice that it was explicit, rather than left to being implicit.

Interviewee 3

So for me, personally, if you see the change [in] the acceptance of equity as a value determining and influencing people's thinking and work, it's good.

Interviewee 5

HIA's usefulness in consolidating knowledge and understanding of health issues and potential health impacts has been noted in the literature [72,75], and approaches that explicitly examine health equity impacts seem likely to enhance understandings of health equity as well.

A challenge that was identified by the interviewees from both the EFHIA working group and the Department of Health was that many of the potential health equity issues that could have arisen did not relate to the overall structure or nature of the ABHI initiatives, but to the way the initiatives would be implemented.

...when I was going through the [EFHIA] recommendations, that some of them appeared to have gone beyond just saying what would happen. If you're just trying to provide equity focused [recommendations], they're often about good planning, which I think was probably very apparent to the people during the equity focused HIA going 'what is the good planning in this?'

Interviewee 1

There were also some things in [the EFHIA report] that, I guess, implied, that we wouldn't consider, some issues that I think can be dealt with in careful planning, and careful implementation, and the intention, as I said before, if the [ABHI implementation plan] was really about 'this is the flavour of where we're going with this' we're going to have to obviously have greater implementation plans around each of these strategies, we've only got sixty pages to do it in.

Interviewee 3

This highlights some of the challenges in undertaking assessments of implementation plans. Policies, such as the ABHI as a whole, are necessarily aspirational, setting out areas of activity in broad terms. EFHIAs, and HIAs in general, need to consider implementation as it is at that stage where many unintended and previously unidentified impacts are likely to arise. The NSW ABHI implementation plan included details of how the initiatives would be implemented, but many of the very detailed planning and implementation activities were appropriately determined by operational and service managers. This identifies a tension relevant to all HIAs, but to EFHIA in particular; to what extent should an HIA focus on making recommendations to assist implementation?

Two preconditions seem to be important enablers of EFHIAs of implementation plans: a high degree of understanding of the policy context and processes being assessed; and trust and constructive engagement between the assessors and those responsible for the development and implementation of the implementation plan. This is similar to the findings of studies that have looked at the impacts of HIAs in the Netherlands [76] and impact assessments more broadly [77].

##### 3. Enabling discussion of alternatives

Several interviewees stated that the EFHIA enabled consideration of different ways of achieving the implementation plan's objectives.

...even during the time we were doing it... we were able to enter into some discussions about what might be alternatives. So I think that in these sorts of environments, we've got an opportunity to influence the implementation. It's actually really important to have debate and that's what I think the EFHIA allowed.

Interviewee 2

The identification and assessment of alternatives is an important and under-emphasised part of HIA and impact assessment practice [13,78,79]. The development of more formal procedures for generating alternatives that address health inequities, which may then be assessed using EFHIA, would be of use in ensuring health equity is considered earlier in the formulation of policy options.

##### 4. Missed opportunities

There was a degree of ambivalence towards the rapid EFHIA on the part of several of the interviewees, amongst both people from the Department of Health and the EFHIA working group. The terms "lost opportunity" or "missed opportunity" came up several times during the interviews. Whilst all five interviewees acknowledged that the EFHIA had some degree of direct and indirect impact on subsequent activities, three interviewees expressed disappointment that more didn't come from doing the EFHIA, in the form of either more robust consideration of equity in health policy in general or ongoing collaboration with the expert panel.

No, it was really a lost opportunity, I think, to get people engaged, and not only engaged in HIA, but in equity...

Interviewee 1

I think there might have been a missed opportunity... The EFHIA focused too much on issues that would have been addressed at later stages in the planning anyway.

Interviewee 4

This can be attributed, at least in part, to feelings that the Department was not able to be fully involved in the EFHIA due to the competing pressures involved in finalising the implementation plan. The EFHIA was viewed by two of the interviewees as being overly critical of the development of the implementation plan and failing to recognise the time-pressured and politicised context it was being developed in.

...by doing an HIA, if you start then telling people how to do good planning, it's almost like it's a little bit insulting to those who believe they are good planners, rightly or wrongly... So I think there's a fine line between telling people how to suck eggs, when they already know how to suck eggs, but doing it in a different way.

Interviewee 3

I think people felt when recommendations came in, that they saw as a critique, or not that they were a critique, because different... They were like 'Oh, but it wasn't a proper plan anyway, it was just, you know, we were just trying to get the money, and that was our goal at that time, just get the money, and we said we'd do this, but not sure if we really will'.

Interviewee 1

This suggests that the involvement of stakeholders and decision-makers in the process of EFHIA is more than an ideological commitment to participation and representation; it is critical in enabling it to have an impact on decision-making and implementation [80,81]. There are of course significant, and possibly insurmountable, tensions between the rapid processes required if EFHIA is to inform policy development and implementation in a timely fashion and the need to engage stakeholders fully in the process of conducting the EFHIA.

##### 5. Differing conceptualisations of the purpose of the EFHIA

Although all participants described changes that were attributable to the EFHIA, in general the Department of Health staff described the EFHIA as making a more modest contribution to the development and implementation of the implementation plan than the EFHIA working group did. This difference may be attributed to greater involvement and familiarity with the ongoing development and roll-out of the implementation plan or differing understandings about the role and purpose of the EFHIA.

We didn't have a shared understanding of why we were undertaking it. Our purposes were probably different from CHETRE's purposes, and maybe that's where they don't work, but if you have two differing purposes, unless you can fully appreciate what those two different purposes are, maybe it doesn't work out as well as it could...

...I think there was a feeling that, well, we could get something out of [the EFHIA]. There were probably two rationales for why it would be useful. One is that we could get some, a critique if you like, or some feedback about, through an equity lens, on the strategies that we had proposed. And the second one was that it would perhaps serve a process of helping people who are more engaged in the consultation process.

Interviewee 3

In a way, it was about improving the quality of the document, it was actually quite important to be able to debate some of the issues.

Interviewee 2

These differences may be partly due to variations in the way people involved in the EFHIA understood the purpose of the HIA in general. Those from the Department of Health tended to describe HIA as a process for using evidence to informing decision-making, whereas those from the EFHIA working group tended to describe HIA as a process for quality enhancement and examining unanticipated impacts.

There is an increasing consensus internationally that impact assessment should be understood as a learning activity [16,82-85]. Glasbergen [86] describes three types of learning that can occur through impact assessment:

• *Technical learning*, which involves searching for technical solutions to fixed policy objectives;

• *Conceptual learning*, which involves redefining policy goals, problem definitions and strategies; and

• Social learning, which emphasises dialogue and increased interaction between policy actors (this is distinct from the concept of social learning described in the psychology literature [87]).

Some of the differences in this case may be understood as different attitudes to desired learning goals of the EFHIA. Many of those from the Department of Health described the EFHIA as a technical learning activity; those involved in the working group described the EFHIA in terms more consistent with conceptual learning. There was very little discussion of impacts that might be classed as social learning within the impact evaluation interviews. Understanding the different types of learning that may come from an EFHIA, and which one is desired within a specific context, is important as different expectations may serve to create confusion and tension amongst those involved. In our work we would describe HIA as both a technical tool and a process and it is this process that provides the opportunities for conceptual and social learning to build ongoing relationships with other stakeholders.

#### Resource description findings

Costs and time details are included in this paper to transparently report what resources were used to undertake the rapid EFHIA and to assist future cost utility analyses [10]. The resources invested were estimated by the EFHIA working group and are detailed in Table 4.

**Table 4:** Estimation of resources invested to undertake the EFHIA

Resource	Project Hours (If Applicable)	Estimated Cost/Hour (If Applicable, USD)	Cost Estimate (USD)
**EFHIA Workshop**	42 hrs (Includes Participants)	$34.80	$1,461.60

**Report Writing**	48 hrs	$34.80	$1,670.40

**Review and Comment on Report**	8 hrs	$34.80	$278.40

**Report Formatting, Referencing and Proof Reading**	8 hrs	$34.80	$278.40

**Travel Costs (1 Airfare)**	-	-	$227.00

**Catering for Workshop**	-	-	$120.00

**TOTAL**	**106**	-	**US $4,035.80**

In Kind Subtotal*	*106*	-	*US $3,688.80*

***Cash Subtotal***	-	-	*US $347.00*

* In-kind costs refers to people's time that was donated, rather than being paid for directly to undertake the EFHIA

The limited number of papers describing the human resource investments made suggest that between 684 and 3,784 project hours for an HIA are not uncommon [88,89]. This suggests that the estimated 106 project hours for this EFHIA was little by comparison.

Costs are also rarely described in the literature but 15 HIAs conducted in Europe have been reported to range between US $1,316 and US $190,878 [73], 15 English HIAs included in a cost benefit study ranged between US $1,578 and US $93,006 [74], and the *Merseyside Guidelines for Health Impact Assessment *reported in 2000 that the mean cost of three HIAs conducted in Liverpool was US $18,033 [90]. This suggests that at US $4,025.80 the EFHIA of the ABHI implantation plan is one of the least costly HIAs that has been documented.

## Limitations

This is paper describes a rapid, specific and contextually situated EFHIA. Care should be taken not to over-generalise the findings to other settings especially as it was conducted in response to the needs of a specific decision-making context.

A limitation in how the EFHIA was conducted was that the rapid EFHIA process relied on expert opinion from a relatively small group. Consultation was limited, as was systematic review of the literature, both largely due to time constraints. There was limited reference to increased dialogue or increased interaction between those involved following the EFHIA. This may suggest that social learning [86] through this EFHIA was limited. This could be due to its rapid nature, though other factors that were not identified may also have limited the extent of further collaboration.

There were also limitations in terms of how the EFHIA's impact was evaluated. Firstly, unlike some other HIAs whose impact has been evaluated [72] there was unfortunately no final document against which recommendations from this EFHIA could be checked off. This is often a feature of higher-level government implementation plans that cross a number of portfolio areas. This is a limitation that should be borne in mind when considering the EFHIA's direct impacts on decision-making. Secondly, a relatively small number of people were interviewed (five) for the impact evaluation. This is because this is the number of people who were intimately involved in both the EFHIA and the further development of the policy implementation plan was limited. This was in part due to the EFHIA's rapid nature, partly due to context-specific practices, i.e. who is involved in the development of policy implementation plans in NSW. Thirdly, several of this paper's authors were involved in conducting the EFHIA. The second, third and fourth authors played an active role (see section on Authors' Contributions). Whilst efforts have been made to ensure that the findings are empirically supported, their involvement in the EFHIA process may have influenced the interpretation of findings. Lastly there is a possibility of recall bias, as some of those interviewed may have revisited the recommendations more often than others interviewed [91].

It is important to note that this paper is not solely an impact evaluation of a rapid EFHIA; it also seeks to describe the methods by which the EFHIA was conducted in some detail. This is because there are relatively few examples of EFHIAs reported in the literature to date, something that is required given the World Health Organization's recent calls for the use of health equity impact assessment [4]. Despite the limitations outlined, measures were taken to ensure procedural fidelity to EFHIA guidance [46] and the features of the impact evaluation interviews are outlined in Table 4.

## Conclusions

Although it was only a rapid process this EFHIA had an impact on the development of the implementation plan. The EFHIA was well received by the Department of Health and its recommendations were incorporated into the NSW ABHI implementation plan's revision. Those responsible for developing specific sections of the implementation plan were asked to demonstrate how they had addressed issues raised in the EFHIA in their section.

This rapid EFHIA process relied to a large extent on expert opinion from a small group of people. There was little capacity to consult with other stakeholders or to systematically review the literature. Despite this the EFHIA has had an impact on the ways in which the ABHI initiatives were planned. This process also highlighted that in many areas, even if there had been more time for a detailed assessment, there was little direct evidence relating to potential inequities or on effective interventions to prevent or redress them [4]. The ability to adapt existing knowledge to new contexts will be an important skill required in future rapid EFHIAs.

This EFHIA has demonstrated that HIA processes can be used within the political realities and time frames within which policy-makers operate. It demonstrated that EFHIA specifically, and HIA generally, can make a contribution to the implementation of health sector initiatives, not just other's sectors decision-making. It was also highlighted as a example of action towards enhanced capacity for monitoring, research, and intervention in the *Final Report of the World Health Organization Commission on the Social Determinants of Health *[4].

This process would not have been possible without the support of NSW Department of Health and the willingness of those involved in the development of the health promotion components of the NSW Department of Health *Australian Better Health Initiative (ABHI) Implementation Plan *to have their work scrutinised by people who largely worked outside the Department. It was also feasible to undertake the assessment within the time constraints due to the involvement of an expert panel with knowledge of the policy area and the ways in which the health system operated. Because this group of people were also experienced in working with government policy processes they were also able to concentrate on how potential problems could be minimised and potential gains enhanced within resource constraints.

It is important that whilst EFHIA can have impacts on decision-making and planning that it not be regarded as a panacea. The evidence that informed the EFHIA was limited and the assessment itself was not comprehensive, though nor did it claim to be. There is a need to be realistic about the extent to which a rapid process can be expected to systematically inform subsequent activities. Given that many policies require considerable time, expertise and resources to develop, however, an investment of four days to ensure that health equity issues have been explicitly considered may be regarded as time well spent.

A major challenge for all HIAs is to be able to respond in flexible and timely ways to the needs of policy-makers who are often developing proposals within brief timeframes and in politicised contexts. A rapid EFHIA process may provide a practical mechanism for looking at the potential health equity impacts of proposed initiatives.

## Abbreviations

ABHI: Australian Better Health Initiative; CHETRE: Centre for Health Equity Training, Research and Evaluation; COAG: Council of Australian Governments; EFHIA: Equity focused health impact assessment; EqIA: Equality impact assessment; HEIA: Health equity impact assessment; HIA: Health impact assessment; NSW: New South Wales.

## Competing interests

The authors declare that they have no competing interests.

## Authors' contributions

BHR contributed to the conceptual development of the article, undertook the impact evaluation and drafted the final version of the article. PH was involved in the EFHIA working group, undertook the process evaluation and contributed to drafts of the article. EH conceived the EFHIA, was involved in the EFHIA working group, conceived the article and contributed to subsequent drafts. LK was involved in the EFHIA working group and contributed to drafts of the article. All authors viewed and approved the final version of the article.

## Supplementary Material

Additional file 1 Rapid Equity Focused Health Impact Assessment of the Australian Better Health Initiative Report Click here for file
