# Supplementary material for: A rapid equity focused health impact assessment of a policy implementation plan: An Australian case study and impact evaluation
Source: Int J Equity Health. 2011 Jan 30;10:6. doi: 10.1186/1475-9276-10-6 (PMC3038149; doi:10.1186/1475-9276-10-6)
Supplement: Additional file 1 — Rapid Equity Focused Health Impact Assessment of the Australian Better Health Initiative Report [file 1475-9276-10-6-S1.PDF]

UNSW
THE UNIVERSITY OF NEW SOUTH WALES

Rapid Equity Focused Health Impact Assessment of the Australian Better Health Intitiative

Assessing the NSW Components of Priorities 1 and 3

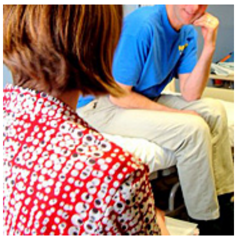
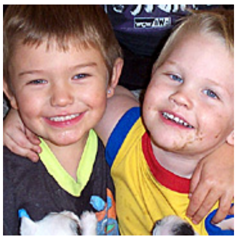
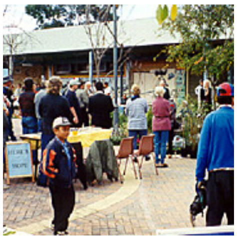
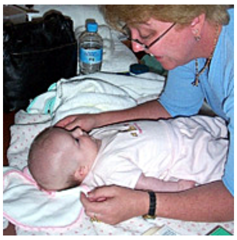
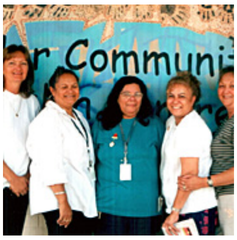
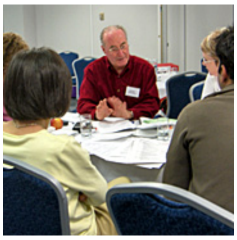
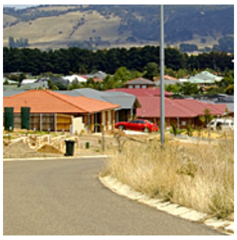
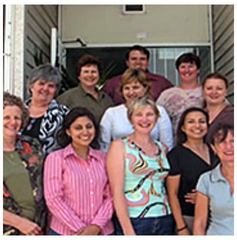
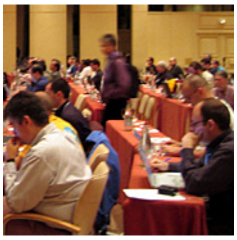

November 2006

UNSW Research Centre for Primary Health Care & Equity

Research that makes a difference

Citation

Elizabeth Harris, Patrick Harris, Lynn Kemp. *Rapid Equity Focused Health Impact Assessment of the Australia Better Health Initiative: Assessing the NSW components of priorities 1 and 3*, UNSW Research Centre for Primary Health Care and Equity: Sydney, November 2006.

Acknowledgments

Michelle Bonner, Mark Harris, Ben Harris-Roxas, Lesley King, Rachel Laws,
Nidia Raya Martinez, Dave Trudinger

Further information

Patrick Harris
Health Inequalities, Health Impact Assessment and Healthy Public Policy Program (CHETRE)
UNSW Research Centre for Primary Health Care & Equity
School of Public Health and Community Medicine, University of New South Wales

Phone +61 2 9828 6230
Fax +61 2 9828 6232

Email patrick.harris@unsw.edu.au

Web http://www.hiaconnect.edu.au

Postal Address
CHETRE LMB 7103
Liverpool BC NSW 1871 Australia

TABLE OF CONTENTS

Part 1: Executive Summary ... 1

Part 2: Introduction ... 6

2.1 Introduction ... 6
2.2 Rationale ... 6
2.3 Scope ... 6
2.4 Summary of the Process and its Limitations ... 7
2.5 Structure of the Report ... 7

Part 3: Summary of Findings ... 8

3.1 Major Issues to Emerge ... 8
3.2 Equity Filters ... 9
Initiative: Urban Health ... 10
Initiative: Child Care Settings ... 11
Initiative: Child Care Settings ... 12
Initiative: Fresh Tastes at School ... 13
Initiative: External Physical Activity Programs ... 14
Initiative: Innovation & Enhancement Grants ... 15
Initiative: Older People’s Physical Activity Opportunities ... 16
Initiative: Lifestyle Interventions for High Risk Diabetes ... 17
Initiative: Healthy lifestyle and Risk Modification Information Service Pilot ... 18
Initiative: Community Health Centre Risk Factor Management ... 19

Part 4: The EFHIA Process ... 20

4.1 What is equity focused HIA? ... 20
4.2 Screening ... 21
4.3 Scoping ... 22
4.4 Identification and Assessment of the potential health impacts ... 24
4.5 Process for developing recommendations ... 24
4.6 Monitoring and Evaluation ... 25
4.7 Reflections on the process ... 25
4.8 References ... 26

Appendix 1: Commissioning Brief ... 27

PART 1: EXECUTIVE SUMMARY

1.1 Background to the Equity Focused Health Impact Assessment

The Australian Better Health Initiative (ABHI) Implementation Plan has been developed as part of a Council of Australian Governments Reform Package aimed at achieving better health for all Australians. In NSW Approximately $20 million of new money has been allocated from Treasury for Priority Areas 1 & 3 of *The ABHI Implementation Plan: Promoting healthy lifestyles and supporting lifestyle and risk modification*.

As part of the development of the ABHI Implementation Plan for NSW, NSW Health asked the UNSW Research Centre for Primary Health Care and Equity to conduct a Rapid Equity Focussed Health Impact Assessment (EFHIA) to:

- make practical recommendations in order to improve the equity of proposed strategies; and
- ensure that the strategies do not unintentionally widen the equity gap.

1.2 Overview of the Equity Focused Health Impact Assessment

The EFHIA focussed on five areas[1] where there are currently health inequalities or potential for health inequalities to develop: age, gender, place of residence, ethnicity and socioeconomic status.

The EFHIA followed the established steps in Health Impact Assessment: screening, scoping, identification and assessment, decision-making and recommendations, and monitoring and evaluation. To streamline the process to meet the required limited timeframe a six hour workshop was organised with six experts in the areas of equity, HIA, health promotion and policy analysis.

During the workshop eight specific initiatives (see recommendations below) from priority areas 1 and 3 of the ABHI implementation plan were assessed using an equity filter based on five questions:

1. What is the initiative trying to do?
2. Is there evidence of inequality?
3. Who may be disadvantaged by the initiative?
4. Are there likely to be unanticipated impacts?
5. What are the key recommendations for implementation?

The EFHIA produced ten general summary recommendations and additional recommendations across the eight specific initiatives.

1.3 Equity Focused Health Impact Assessment Summary Recommendations

- An explicit statement on the purpose of the initiative being to improve the health of all people in NSW while reducing the gap in health and health risk between those who are most and least advantaged should be included in the introduction.
- The initiative should to be clearly aligned to other existing programs and strategies that focus on the prevention and management of chronic disease. This should be done in ways

[1] While important from an equity perspective the specific needs of Aboriginal people were not considered due to work already conducted by NSW Health as part of the development of the implementation plan in collaboration with the Aboriginal Health Branch in NSW Health.

that strengthen opportunities for addressing equity in existing initiatives and make them more sustainable.

- A set of "core" prevention activities should be identified within the initiative that should be available to all residents of NSW. If this was done (and audited) many issues related to health equity would be addressed. For example social marketing campaigns should be developed that segment the population and ensure that all groups have access to health messages in ways that are culturally appropriate.
- Grants for AHS and general funding for specific program areas should be used to enhance existing initiatives rather than develop additional short term programs that are unsustainable. This enhancement should focus on reorienting these programs if necessary to increase access to those populations that are currently poorly served.
- Those responsible for implementation should be provided with training and support systems (including web-based information) that would give them access to evidence on "best buys" for intervention with disadvantaged groups.
- Community organisations, local communities and consumer should be systematically identified as key partners in all relevant strategies.
- The issue of age is seen as adequately addressed in the document with investment in the early years and middle and older age groups.
- Increased gendered analysis should be undertaken and strategies developed that recognise the differing needs on men and women. This relates to all strategies.
- Increased attention to addressing capacity and resource issues in rural and remote communities is required.
- The evaluation should include routine analysis of the reach of programs into the community and identification of those groups not using services in relation to need.

1.4 Recommendations 1.3.2, 1.3.3, 1.3.4, 1.3.5, 1.3.6, 3.1.1, 3.1.2, & 3.1.4

1.3.2 Chronic disease prevention funding program – local government and urban planning setting

- *Implementation strategy* to include discussions with key stakeholders to differentiate the potential complementary roles of the three initiatives. These potential links to be made more explicit in the implementation plan.
- *Identify opportunities to work with other stakeholders:* A first step is collaborative mapping of key stakeholders involved in urban planning and points of intervention, especially in relation to meeting the needs of disadvantaged communities.
- *Healthy Urban Planning Unit* to be developed in a phased way to ensure greatest reach and effectiveness. Phase 1 would focus on consultation with key stakeholders and consensus building. Phase 2 would look at workforce and organisation development issues. Phase 3 would focus on institutionalising healthy planning processes.
- *Local Government Grants program* to focus explicitly on developing capacity of Local Government to develop Healthy Urban Planning processes. The initial focus of this might be on the development of a stronger focus on health in Social Plans, with a specific focus on the distribution of health and health risk within their population

1.3.3 Chronic disease prevention funding program – child care setting

1.3.3.1 Physical activity guidelines/recommendations and development of policy and resource materials

- *Developing guidelines to include* strategies for the implementation and dissemination to be sustained over a long period of time. Priorities will be given to the location and types of services most used by disadvantaged groups (For example rural communities and family day care services)
- *Scoping exercise to include* mapping the use of children's services in NSW to identify users and groups that may be disadvantaged (for example disadvantaged families using home-based care).
- *Dissemination of the materials to include* differential roll out, with centres in disadvantaged areas and those service types with large numbers of children from priority populations (Rural, Culturally and Linguistically Diverse Populations (CALD), Socioeconomic Position (SEP) the first to be included.
- *Appropriate training of childcare workers through making available* additional funding for institutions in disadvantaged areas for staff to attend training.
- *Additional items:* The guidelines will inform accreditation standards and training programs for staff in child care settings. The evaluation of the guidelines will include whether these have been adopted in disadvantaged areas.

1.3.3.2 Enhancement of the Environmental Tobacco Smoke (ETS) and children campaign

- *Utilise child care settings to investigate* Family Day Care as a key setting where smoking may occur. Further discussion with service providers of Family Day Care organisations to provide support to carers and family members to stop smoking while in the home when children are in care.
- *Community grants* targeted toward disadvantaged areas to address differential uptake and minimal engagement with ETS.
- *Develop training programs* which focus on behaviour that leads to passive smoking rather than stigmatising or stereotyping parents or providers.
- *Utilise opportunity to reinforce this initiative* through linking with the NSW Department of Community Service revision of Health and Safety Guidelines for Family Day Care.

1.3.4 Chronic disease prevention funding program – school setting

1.3.4.1 Enhance the "Fresh Tastes @ School" program

- *"Fresh Tastes @ School" initiative* to identify strategies to encourage the most disadvantaged schools to "opt in" to the program. For example providing support and incentives to teachers who may act as the program champion.
- *"Fresh Tastes @ School" program* to provide additional funding to schools classified as disadvantaged according to school index (similar to established Department of Education and Training fund).
- *Key partners* to include parents and students.

1.3.4.2 Implement an externally provided physical activity programs in school and after-school settings

- *Investigation of quality to map* the range of external programs offered and their use according to potentially disadvantaged population groups at both state and/or local levels.
- *Minimum standards* to establish a system for accreditation of programs that includes an assessment of gender and cultural competence in their delivery.
- *Additional item:* Include monitoring investment in external programs within disadvantaged schools to build an evidence base for potential equity impacts.

1.3.5 Chronic disease prevention funding program – community setting

- *'health services'* as a setting requires clearer definition as a setting to improve equity.
- *The funding model* to give preference to enhancing existing strategies and programs in order to improve targeting of disadvantaged groups and populations, for example CALD and diabetes prevention programs.
- *AHS grants* to be supported by NSW Health to develop a better understanding of where the most equitable programs and activities are best positioned across all SNAP risk factors and four settings, for example investment in the early years for disadvantaged populations.
- *Evaluation component* to be systematic to determine whether the funding may have been more usefully used to build infrastructure and capacity within AHS and partner organisations to undertake and extend existing equitable programs rather than develop new programs.
- *Additional item:* level of funding to be committed by AHS requires clarity.

1.3.6 Physical activity provider capacity building project

- *Community provider partnerships* to focus on communities where there are few private providers but high levels of need to develop new approaches to program delivery. For example partnerships with neighbourhood centre activities and local clubs.
- *Public private partnerships* to include incentives for private providers to participate will be important, especially in areas where it may take time to develop a client base.
- *1800 number* to include consideration of access by CALD and culturally and linguistically appropriate advice early in the planning stage.
- *Additional item:* Transport to programs and accessible venues will be an issue for many older people. This initiative can be linked with initiative 3.1.2 to provide an integrated information service.

3.1.1 Community based diabetes prevention program - pilot

- *Focus of initiatives* requires clarity regarding the individual service delivery focus or the ecological model of the Finnish program, as disadvantaged communities are more likely to benefit from a more ecological and integrated approach.
- *Network with other jurisdictions* to access and share learnings about diabetes prevention programs conducted throughout Australia.
- *Within AHS the focus on priority populations* should be on areas where there are sizeable pockets of disadvantage. Focussing the program across an entire AHS is unlikely to be able to achieve change.

- *Assessment advice and referral* to be based on referral points that have low or no cost, and timely access (that is, short or no waiting times).
- *An analysis of barriers* to screening for high risk (pre diabetes) and referral should be conducted for low SES groups in each locality.
- *Establishing goals* requires the use of bi-lingual educators

3.1.2 Lifestyle and risk factor modification information service

- *The pilot* to include a strong evaluative component: who uses the service, how similar or different it is from the Quitline, including how it arranges referral to other services, and the impact on other services including unmet demand.
- *Positioning the service* to address access by CALD and isolated rural communities and ensuring the quality of service provided by any subcontracted Non-Government Organisations (NGOs)for specific groups (eg CALD)
- *Enhance information systems* to develop and maintain links to local services as a referral base

3.1.4 Risk factor assessment and management project

- *Information system* to improve understanding of work currently being done and how this may need to be developed or re-shaped over time to effectively engage in disadvantaged groups. This will include an understanding of the reach of activities and groups using services.
- *Training and resources* to be assessed for cultural appropriateness and suitable literacy levels.
- *Local service development* to be based on development of general principles and programs to avoid a proliferation of programs that are difficult to evaluate. These principles should include strategies for including disadvantaged groups, including community engagement.
- *Linkage with general practice programs* to optimally use health care resources in disadvantaged communities.
- *Additional item:* This initiative to be explicitly integrated into other chronic disease initiatives (particularly 3.1.2, 3.1.5, 1.3.6, 1.3.5)

PART 2: INTRODUCTION

2.1 INTRODUCTION

This report presents the findings of a rapid equity focused Health Impact Assessment (EFHIA) that was undertaken on the Australian Better Health Initiative (ABHI) Implementation Plan (Draft dated 13th November). Its aim was to identify ways in which the implementation plan could have a stronger emphasis on addressing health inequalities in NSW.

The Australian Better Health Initiative is aimed at achieving better health for all Australians. This initiative provides for the implementation of a range of activities promoting good health and reducing the impact of chronic disease. It focuses on a number of priority areas, of particular relevance to the Centre for Chronic Disease Prevention and Health Advancement are two priority areas:

1. Promoting healthy lifestyles;
3. Supporting lifestyle and risk modification.

The NSW Government has committed $20 million, from mid 2006 for a four-year period to support the implementation of the Australian Better Health Initiative in NSW across these two priority areas. Implementing a range of initiatives within these areas will build on the considerable work already being implemented across the state, aimed at reducing the harm associated with a number of chronic diseases, including:

- The NSW Tobacco Action Plan;
- Prevention of Obesity in Children and Young People: The NSW Government Action Plan 2003 - 2007;
- NSW Falls Policy; and
- NSW Chronic Disease Prevention Strategy 2003 – 2007

As part of the consultation process on the ABHI Implementation Plan the Centre for Health Equity Training, Research and Evaluation (UNSW) was asked to comment on the proposal. Following discussions with NSW Health it was decided to undertake a Rapid EFHIA to improve the equity focus of the ABHI Implementation Plan on eight initiatives under the two priority areas.

2.2 RATIONALE

The burden of chronic disease is in the Australian community has a strong social gradient with those people who are least advantaged having higher levels of mortality, morbidity and risk than those who are most advantaged. The sizes of these differences are large and have implications for service delivery as well as for primary, secondary and tertiary prevention programs. For example for every thousand people with diabetes in the most advantaged areas of NSW there are 1,800 people in the most disadvantaged areas. This is also reflected in patterns of health risk behaviour. The recent Australian Institute of Health and Welfare (AIHW) reports on Chronic Disease and Health Inequity as well as the NSW Chief Health Officer (CHO) Report provide detailed information on these patterns related to age, gender, ethnicity and place of residence.

It is also recognised that may disadvantaged groups do not benefits from many of the traditional health promotion approaches and if this is not addressed then there is the potential for many health promotion interventions to increase health inequity.

2.3 SCOPE

NSW Health agreed to a rapid EFHIA on eight of the strategies within the initiative (1.3.2-1.3.6 and 3.1.1, 3.1.2, and 3.1.4 – see Appendix One). Given the nature of the implementation plan being top line in nature, it was determined that the purpose of the EFHIA was to provide practical recommendations in relation to the implementation and roll out of some of the proposed ABHI initiatives based on an equity filter being applied to the initiative descriptions.

During scoping, given the short timeframe for the EFHIA, CHETRE determined the EFHIA was not to address issues related to Aboriginal populations as this was being negotiated with the Aboriginal Health Branch. This was agreed to by the steering committee. The steering committee agreed to focus on five areas where there are currently health inequalities or potential for health inequalities to develop: age, gender, place of residence, ethnicity and socioeconomic position.

2.4 SUMMARY OF THE PROCESS AND ITS LIMITATIONS

As the potential health impacts of the ABHI Initiatives are potentially large and the level of investment is high, normally a minimum of a 6-12 week period would be recommended to undertake the HIA. However in order to be able to contribute to the policy process within a tight time frame it was decided to undertake a rapid EFHIA based on the use of an equity filter. This involved a 6 hour workshop and two teleconferences with the Steering Committee. As part of the screening and scoping process (See Part 3) we agreed on an equity filter that asked five questions:

1. What is the initiative trying to do?
2. Is there evidence of inequality?
3. Who may be disadvantaged by the initiative?
4. Are there likely to be unanticipated impacts?
5. What are the key recommendations for implementation?

This work was undertaken over four working days by the equivalent of 2 equivalent full-time staff members and needed to fit with other commitments. Because of the time constraints we were limited in:

- the number and range of people who could be involved in the Steering Committee;
- the level of external consultation that could be undertaken, especially with the population groups that has been identified; and
- accessing directly relevant evidence of patterns of health inequity for each strategy.

The process was largely informed by expert opinion and the key documents that were used (See Section 3). This means that some of the analysis is somewhat superficial and is unable to unpick some of the complexity in looking at each of the population groups, for example patterns of health risk within and between CALD communities is not homogeneous.

Also because this is the first time that any of those involved had been involved in such a rapid appraisal we needed to identify processes for gathering and presenting the findings that adequately reflected the intentions of the original strategies.

2.5 STRUCTURE OF THE REPORT

This report has four parts:

Part 1: Provides an executive summary of the EFHIA

Part 2: Provides an overview of the rationale for conducting the Rapid EFHIA, an outline of how it was conducted and identifies the limitations of the process.

Part 3: Presents a summary of the findings of the EFHIA and detailed commentary on each of the strategies using an equity screen.

Part 4: Describes the process through which the EFHIA was undertaken in more detail.

PART 3: SUMMARY OF FINDINGS

In this section of the report provides an overall summary of the major themes to emerge through the EFHIA and the results of the equity filter on the eight strategies is presented. Some of the issues that emerged are general and relate to the broad directions of the initiatives while others relate directly to issues of equity. This reflects an understanding that both mainstream and targeted approaches are needed to reducing health inequity. A well functioning and integrated health system, including health promotion initiatives, make a major contribution to health improvement for all parts of the population. In addition it may also be necessary to target and modify strategies to make them more relevant and accessible to specific population groups.

3.1 MAJOR ISSUES TO EMERGE

The Rapid Equity focussed HIA has identified a number of ways in which the proposal should be strengthened to increase the initiatives equity focus.

1. An explicit statement on the purpose of the initiative being to improve the health of all people in NSW while reducing the gap in health and health risk between those who are most and least advantaged should be included in the introduction.
2. The initiative should to be clearly aligned to other existing programs and strategies that focus on the prevention and management of chronic disease. This should be done in ways that strengthen opportunities for addressing equity in existing initiatives and make them more sustainable.
3. A set of “core” prevention activities should be identified within the initiative that should be available to all residents of NSW. If this was done (and audited) many issues related to health equity would be addressed. For example social marketing campaigns should be developed that segment the population and ensure that all groups have access to health messages in ways that are culturally appropriate.
4. Grants for AHS and general funding for specific program areas should be used to enhance existing initiatives rather than develop additional short term programs that are unsustainable. This enhancement should focus on reorienting these programs if necessary to increase access to those populations that are currently poorly served.
5. Those responsible for implementation should be provided with training and support systems (including web-based information) that would give them access to evidence on “best buys” for intervention with disadvantaged groups.
6. Community organisations, local communities and consumer should be systematically identified as key partners in all relevant strategies.
7. The issue of age is seen as adequately addressed in the document with investment in the early years and middle and older age groups.
8. Increased gendered analysis should be undertaken and strategies developed that recognise the differing needs of men and women. This relates to all strategies.
9. Increased attention to addressing capacity and resource issues in rural and remote communities is required.
10. The evaluation should include routine analysis of the reach of programs into the community and identification of those groups not using services in relation to need.

3.2 EQUITY FILTERS

The results of the equity filter on each of the initiatives are presented. As well there is an assessment on the likely size of impact of the strategy on improving reducing the risk of chronic disease (High, medium, small and negligible) and the likelihood of this impact being realised (Definite, Probable and Speculative). These were assessments made by the Steering Committee based on their understanding of the issues and strategies.

Population groups identified in the strategy are identified in the table and those added as the result of the equity filter are added.

The eight strategies covered in this document are:

1.3.2 Healthy Urban Planning

1.3.3 Physical Activity and healthy eating in child care settings

1.3.4 Enhance the “Fresh Tastes @ School” Program

1.3.5 Innovation and enhancement grants

1.3.6 Physical activity provider capacity building project

3.1.1 Community based diabetes prevention program –pilot

3.1.2 Lifestyle and risk factor modification information service

3.1.4 Risk factor assessment and management project

INITIATIVE: URBAN HEALTH

1.3.2 Chronic disease prevention funding program – local government and urban planning setting

Potential Size of Impact: Large
Likelihood of Impact: Probable
Populations Affected:

	Whole of Population	Age	Gender	Socio-Economic Position	Ethnicity	Place of Residence
**Identified in Proposal**	✓					
**Added in Appraisal**	✓			✓		✓

What is the initiative trying to do?
The initiative aims to influence the creation of healthy and sustainable urban environments in NSW. Three approaches to achieving this are proposed: developing an infrastructure for Healthy Urban planning, designing places for active living, and local government grants. System change is acknowledged and the use of appropriate tools for healthy urban planning are noted. The three approaches proposed have considerable overlap but collectively have the potential to improve health through the creation of healthy and sustainable environments.

Is there evidence of inequality?
There is evidence that current urban planning and development processes are resulting in the development of communities that have poor access to infrastructure that is important for health outcomes such as access to public transport, walkable neighbourhoods or access to services. These structural processes are often invisible to individuals but play a key role in determining opportunities for health.

Who may be disadvantaged by the initiative?
The current focus on green-field or in-fill development may mean that most of the urban form which already exists in Sydney will be unaffected. The grants process may overburden local government in resource poor areas and be unsuccessful.

Unanticipated impacts?
If these three initiatives are not linked there is the potential for them all to be trying to engage agencies such as Department of Planning, Local Government in what may appear to be competing programs. There is also a danger that the grants program may not “value add” to the overall aims of the initiative but lead to the development of a range of small scale, unsustainable programs.

Equity recommendations for implementation
- *Implementation strategy* to include discussions with key stakeholders to differentiate the potential complementary roles of the three initiatives. These potential links to be made more explicit in the implementation plan.
- *Identify opportunities to work with other stakeholders:* A first step is collaborative mapping of key stakeholders involved in urban planning and points of intervention, especially in relation to meeting the needs of disadvantaged communities.
- *Healthy Urban Planning Unit* to be developed in a phased way to ensure greatest reach and effectiveness. Phase 1 would focus on consultation with key stakeholders and consensus building. Phase 2 would look at workforce and organisation development issues. Phase 3 would focus on institutionalising healthy planning processes.
- *Local Government Grants program* to focus explicitly on developing capacity of Local Government to develop Healthy Urban Planning processes. The initial focus of this might be on the development of a stronger focus on health in Social Plans, with a specific focus on the distribution of health and health risk within their population.

INITIATIVE: CHILD CARE SETTINGS

1.3.3 Chronic disease prevention funding program – child care setting

Physical Activity Guidelines/Recommendations and development of policy and resource materials for the promotion of healthy eating and physical activity in childcare settings for 2 – 5 year olds

Potential Size of Impact: Large

Likelihood of Impact: Speculative as dependent on the effectiveness of dissemination

Populations Affected:

	Whole of Population	Age	Gender	Socio-Economic Position	Ethnicity	Place of Residence
**Identified in Proposal**		✓				
**Added in Appraisal**				✓	✓	✓

What is the initiative trying to do?

The initiative recognises that a large number of children in NSW spend at least part of the week in formal child care settings. Some children spend extended hours in child care. The development of physical activity and nutrition guidelines has the potential to have a positive impact on the health of these children and contribute to the prevention of chronic disease later in life. This initiative is congruent with the core business of children's services

Is there evidence of inequality?

There is some evidence that children in disadvantaged areas and rural and remote areas have poorer access to quality child care services. The Committee felt that mapping the use of child care services needs to be a first step in implementation.

Who may be disadvantaged by the initiative?

Children living in rural and remote areas where there are few services and those children whose parents are unable to afford services or reach them.

Unanticipated impacts?

The guidelines may be differentially taken up by children's' services providers with a chance that those services that are part of larger organisations, or with wealthier parents, being able to adopt the guidelines more easily. There may be a greater impact on children in long day care. The guidelines may impact on the cost of food provided by child care services.

Equity recommendations for implementation

- *Developing guidelines to include* strategies for the implementation and dissemination to be sustained over a long period of time. Priorities will be given to the location and types of services most used by disadvantaged groups (For example rural communities and family day care services)
- *Scoping exercise to include* mapping the use of children's services in NSW to identify users and groups that may be disadvantaged (for example disadvantaged families using home-based care).
- *Dissemination of the materials to include* differential roll out, with centres in disadvantaged areas and those service types with large numbers of children from priority populations (Rural, CALD, SEP) the first to be included.
- *Appropriate training of childcare workers through making available* additional funding for institutions in disadvantaged areas for staff to attend training.
- *Additional items:* The guidelines will inform accreditation standards and training programs for staff in child care settings and the evaluation of the guidelines will include whether these have been adopted in disadvantaged areas.

INITIATIVE: CHILD CARE SETTINGS

1.3.3 Chronic disease prevention funding program – child care setting

Enhancement of the Environmental Tobacco Smoke (ETS) and Children campaign

Potential Size of Impact: Large

Likelihood of Impact: Speculative as dependent on the effectiveness of dissmenination

Populations Affected:

	Whole of Population	Age	Gender	Socio-Economic Position	Ethnicity	Place of Residence
**Identified in Proposal**		✓				
**Added in Appraisal**				✓	✓	✓

What is the initiative trying to do?

The initiative is attempting to use child care settings as a way of making contact with large number of parents in support of the ETS campaign.

Is there evidence of inequality?

There is strong evidence of a socioeconomic gradient in smoking. There are high levels of smoking in some CALD communities and in rural and remote communities. There is also evidence that these communities do not respond as well to mass media campaigns as the general population. It will be difficult to ensure there is access to referral and support services in these settings.

Who may be disadvantaged by the initiative?

The Committee felt that providing information on ETS was not “core business” of children’s services which are smoke free. It is unlikely that distribution of pamphlets and other information will reach those in most need.

Unanticipated impacts?

Child care providers will be expected to be involved in activities and programs that may have marginal gain in smoking cessation but also leave less time to focus on core business. There is a risk that smoking parents may be overly stigmatised by the approach and modify their use of child care services.

Equity recommendations for implementation

- *Utilise child care settings to investigate* Family Day Care as a key setting where smoking may occur. Further discussion with service providers of Family Day Care organisations to provide support to carers and family members to stop smoking while in the home when children are in care.
- *Community grants* targeted toward disadvantaged areas to address differential uptake and minimal engagement with ETS.
- *Develop training programs* which focus on behaviour that leads to passive smoking rather than stigmatising or stereotyping parents or providers.
- *Utilise opportunity to reinforce this initiative* through linking with the NSW Department of Community Service revision of Health and Safety Guidelines for Family Day Care.

INITIATIVE: FRESH TASTES AT SCHOOL

1.3.4 Chronic disease prevention funding program – school setting

Enhance the "Fresh Tastes @ School" program

Potential Size of Impact: Large on schools, Medium on diets

Likelihood of Impact: Probable but with heterogenous causes

Populations Affected:

	Whole of Population	Age	Gender	Socio-Economic Position	Ethnicity	Place of Residence
**Identified in Proposal**		✓		✓		
**Added in Appraisal**			✓	✓	✓	✓

What is the initiative trying to do?

The initiative seeks to build on programs that are already underway to increase access to healthy food in school and which have been reported to have positive impacts. Part 2 or 3 of this program is underway and the initiative aims to extend this work to sectors not previously addressed; Catholic and independent school sectors, and a focus on implementation in disadvantaged areas areas, particularly those with a high proportion of Aboriginal and Torres Strait Islander communities.

Is there evidence of inequality?

The infrastructure and capacity requirements for schools to actively participate in these types of programs have been recognised. There is conflicting evidence on the extent to which different population groups are inequitably influenced by lack of access to healthy canteens. However because of risk factors later in life it is important to promote good eating habits early in life.

Who may be disadvantaged?

Who may be disadvantaged depends on how the program is planned and implemented. For example, focussing on catholic and independent schools as generic and homogenous entities may disadvantage those schools that are ethnically diverse. Schools with limited financial capacity may be disadvantaged due to costs of foods and addition food preparation time.

Unanticipated impacts?

While the move to work with Catholic and Independent Schools is positive these are not homogenous sectors and may have differing needs, for example the appropriateness of the program to Muslim Schools. If the program works on an "opt in" scheme there is a risk that those schools with the least capacity to participate will not join. For example there may be limited numbers of teachers and staff, lack of funding for maintenance, and issues with pupils who have less opportunity than others. These issues may result in the "Fresh Tastes at School" program becoming a low priority and these schools not opting in to the program.

Equity recommendations for implementation

- *"Fresh Tastes @ School" initiative* to identify strategies to encourage the most disadvantaged schools to "opt in" to the program. For example providing support and incentives to teachers who may act as the program champion.
- *"Fresh Tastes @ School" program* to provide additional funding to schools classified as disadvantaged according to school index (similar to established Department of Education and Training fund).
- *Key partners to include* parents and students.

INITIATIVE: EXTERNAL PHYSICAL ACTIVITY PROGRAMS

1.3.4 Chronic disease prevention funding program – school setting

Implement an externally provided physical activity programs in school and after-school settings

---

Potential Size of Impact: Large

Likelihood of Impact: Speculative/small

Populations Affected:

	Whole of Population	Age	Gender	Socio-Economic Position	Ethnicity	Place of Residence
**Identified in Proposal**		✓		✓		
**Added in Appraisal**				✓	✓	✓

What is the initiative trying to do?

The initiative recognises the growing number of externally provided physical activity programs in the school and after school setting. The aim is to investigate the quality of such initiatives and provide recommendations about the development of minimum standards. In addition the aim is to work with providers and the school and after school sectors to enhance access to these programs and the targeting of 'physically inactive children and young people' across all socio-economic areas.

Is there evidence of inequality?

It was not known if there were patterns of health inequity in access to these programs but it likely that cost, transport and time will be issues. There may be issues for girls, especially from CALD, in terms of the types of activity that are suitable. Social pressures and environmental factors may also impact on the numbers of inactive children in schools.

Who may be disadvantaged?

Children attending schools who do not have the capacity to participate in the programs, and children from CALD who may find the program culturally inappropriate, may be disadvantaged. Rural children and children attending schools out of their local area who use public transport would have limited access to after school programs.

Unanticipated impacts?

There is no clear definition of quality and this will need to be clearly articulated to measure potential benefits across a range of programs. There is a risk that as more external providers are working in schools and after school settings that infrastructure for physical activity within schools will be weakened, for example less investment in school owned equipment. These programs may also be attracting children who are already active.

Equity recommendations for implementation

- *Investigation of quality to map* the range of external programs offered and their use according to potentially disadvantaged population groups at both state and/or local levels.
- *Minimum standards* to establish a system for accreditation of programs that includes an assessment of gender and cultural competence in their delivery.
- *Additional item:*
    - Include monitoring investment in external programs within disadvantaged schools to build an evidence base for potential equity impacts.

INITIATIVE: INNOVATION & ENHANCEMENT GRANTS

1.3.5 Chronic disease prevention funding program – community setting

Area Health Service Integrated Chronic Disease Prevention Innovation and Enhancement Grants

Potential Size of Impact: Small

Likelihood of Impact: Speculative

Populations Affected:

	Whole of Population	Age	Gender	Socio-Economic Position	Ethnicity	Place of Residence
**Identified in Proposal**		✓				
**Added in Appraisal**						✓

What is the initiative trying to do?

The initiative provides grants to implement a range of chronic disease prevention activities targeting priority populations and priority settings in the community. There is an explicit focus on SNAP across settings of Local Government, Health Service, Schools, and Childcare. AHSs apply for grants of $152,000 for programs targeting local communities. While clarity is required on how much money the AHS need to contribute, if all areas receive the same amount of money rural areas may be positively advantaged.

Is there evidence of inequality?

Within each AHS there will be a range of vulnerable and disadvantaged populations. A major problem with grants programs is that they are short term and often unsustainable. Use of these grants to set up short –term programs in areas where there is unlikely to be ongoing funding can have negative impacts on trust between health services and communities.

Who may be disadvantaged?

If these programs are rolled out across the AHS at a population level ($50pa over 3 years) they are unlikely to have any substantial impact unless linked closely to existing initiatives.

Unanticipated impacts?

There are risks to sustainability associated with innovation. A focus on innovation may divert attention from current initiatives and practices and strategies, and evidence based practice, toward the short term. In particular, smoking and alcohol are the risk factors which make the greatest contribution to health inequalities. Diverting attention to other initiatives may result in greater death rates from smoking and alcohol related diseases in disadvantaged groups. AHSs currently have different capacities to develop and undertake new projects and programs, particularly in rural areas. The costs associated with this work will be increased in rural and remote areas. Alcohol is not mentioned as a risk factor.

Equity recommendations for implementation

- *'health services'* as a setting requires clearer definition as a setting to improve equity.
- *The funding model* to give preference to enhancing existing strategies and programs in order to improve targeting of disadvantaged groups and populations, for example CALD and diabetes prevention programs.
- *AHS grants* to be supported by NSW Health to develop a better understanding of where the most equitable programs and activities are best positioned across all SNAP risk factors and four settings, for example investment in the early years for disadvantaged populations.
- *Evaluation component* to be systematic to determine whether the funding may have been more usefully used to build infrastructure and capacity within AHS and partner organisations to undertake and extend existing equitable programs rather than develop new programs.
- *Additional item:* level of funding to be committed by AHS requires clarity.

INITIATIVE: OLDER PEOPLE'S PHYSICAL ACTIVITY OPPORTUNITIES

1.3.6 Physical Activity Provider Capacity Building Project

Potential Size of Impact: Small

Likelihood of Impact: Speculative

Populations Affected:

	Whole of Population	Age	Gender	Socio-Economic Position	Ethnicity	Place of Residence
**Identified in Proposal**		✓				
**Added in Appraisal**		✓		✓	✓	

What is the initiative trying to do?

The initiative aims to increase the capacity of community-based providers to provide physical activity opportunities for middle aged and older people. Public private partnerships will be established and strengthened. Certification and accreditation opportunities for private providers will be developed with TAFE and Fitness Australia. A 1800 telephone number will provide information about local access to appropriate physical activity programs.

Is there evidence of inequality?

There are less likely to be private providers in rural & remote communities and in disadvantaged areas. Levels of physical activity are lower in some CALD and disadvantaged groups. Time, transport, cost, type of exercise can all act as barriers to participation in physical activity programs. Those with low literacy, low self-esteem, and with limited English language skills tend not to use 1800 numbers.

Who may be disadvantaged by the initiative?

Those in disadvantaged areas where there are no providers, especially rural and remote communities. There will also be issues related to the types of activities that are acceptable for example to CALD groups, and those with mobility and transport difficulties. Cost is likely to be an important issue.

Unanticipated impacts?

This program needs to be integrated with other initiatives such as Healthy Life Scripts and Division of General Practice programs, to identify people who are not exercising as there is a chance that these new programs may attract existing users of other programs. If a broad definition of private sector providers is taken it may provide opportunities for RSL and Bowling Clubs to extend the range of their activities. Reliance on identified physical activity providers may advantage those who already have good access.

Equity recommendations for implementation

- *Community provider partnerships* to focus on communities where there are few private providers but high levels of need to develop new approaches to program delivery. For example partnerships with neighbourhood centre activities and local clubs.
- *Public private partnerships* to include incentives for private providers to participate will be important, especially in areas where it may take time to develop a client base.
- *1800 number* to include consideration of access by CALD and culturally and linguistically appropriate advice early in the planning stage.
- *Additional item:*
  - Transport to programs and accessible venues will be an issue for many older people.
  - This initiative can be linked with initiative 3.1.2 to provide an integrated information service.

INITIATIVE: LIFESTYLE INTERVENTIONS FOR HIGH RISK DIABETES

3.1.1 Community based diabetes prevention program - pilot

Potential Size of Impact: Medium

Likelihood of Impact: Speculative

Populations Affected:

	Whole of Population	Age	Gender	Socio-Economic Position	Ethnicity	Place of Residence
**Identified in Proposal**						✓
**Added in Appraisal**		✓		✓	✓	✓

What is the initiative trying to do?

This strategy aims to develop and evaluate pilot community-based diabetes prevention strategies to increase intensive lifestyle interventions for those at high risk of diabetes. The initiative is based on evidence from programs in Finland and the U.S. It is proposed the pilot operate in two AHSs, one rural and one metropolitan. Interventions are based on supported lifestyle modification program and establishing goals for participants to address the SNAP risk factors. It is not clear how the community component of the program will be implemented as the current focus is currently on working at an individual level.

Is there evidence of inequality?

There is clear evidence of differences in risk related to rurality, disadvantaged communities, and some CALD groups. There is also likely to be system inequity in capacity to deliver these programs in equitable ways. There is a higher prevalence of impaired glucose tolerance in lower socioeconomic communities, but lower rates of both screening and referral of people with pre-diabetes to diet/physical activity programs.

Who may be disadvantaged by the initiative?

Lifestyle interventions disadvantage those with less capacity and resources to access such programs and implement suggested lifestyle changes, who are generally those at greater risk of diabetes. This may be compounded by lack of health workers to undertake assessment and referral tasks. The involvement of private referral services (eg dieticians or physical activity programs) and the threshold cost of these services which will act as a barrier to referral will be more of an issue in disadvantaged communities

Unanticipated impacts

For community initiatives to be fully equitable (especially in reaching the disadvantaged) these need to be within an integrated primary health care framework that links all chronic disease programs with community based initiatives. This ecological approach is not mentioned here and may lead to limited improvements, particularly for more disadvantaged groups.

Equity Recommendations for implementation

- *Focus of initiatives* requires clarity regarding the individual service delivery focus or the ecological model of the Finnish program, as disadvantaged communities are more likely to benefit from a more ecological and integrated approach.
- *Network with other jurisdictions* to access and share learnings about diabetes prevention programs conducted throughout Australia.
- *Within AHS the focus on priority populations* should be on areas where there are sizeable pockets of disadvantage. Focussing the program across an entire AHS is unlikely to be able to achieve change.
- *Assessment advice and referral* to be based on referral points that have low or no cost, and timely access (that is, short or no waiting times).
- *An analysis of barriers* to screening for high risk (pre diabetes) and referral should be conducted for low SES groups in each locality.
- *Establishing goals* requires the use of bi-lingual educators.

INITIATIVE: HEALTHY LIFESTYLE AND RISK MODIFICATION INFORMATION SERVICE PILOT

3.1.2 Lifestyle and risk factor modification information service

Potential Size of Impact: Small

Likelihood of Impact: Speculative

Populations Affected: * Noted in proposal that the initiative may widen the gap in health inequalities.

	Whole of Population	Age	Gender	Socio-Economic Position	Ethnicity	Place of Residence
**Identified in Proposal**	✓*					
**Added in Appraisal**				✓	✓	✓

What is the initiative trying to do?

The initiative aims to pilot an information model based on the Quitline model to provide information on healthy lifestyles and risk factor management through a centralised call-centre. While initially distinct from smoking cessation there is a view to integrate the service across all SNAP risk factors, and an opportunity to ensure that consistent advice on healthy lifestyles is available to all parts of the health system.

Is there evidence of inequality?

There is concern that there may be differential access to Quitline model, and differential uptake of the messages – with lower levels of uptake by low SES and Aboriginal smokers and those of CALD backgrounds. Use of the existing Quitline could act as an indicator of some of the potential difficulties.

Who may be disadvantaged by the initiative?

Those may include: those with limited phone access or reliance on mobile phones, for example the elderly or low SES groups who may be most at risk; those who work non-standard hours. People with limited English may not access the service. It is not clear how the “call back” component may be implemented. For nutrition and physical activity, there will also need to be referral, requiring a process for ensuring accurate and up to date information on local referral services and programs . This is likely to be a more important issue than for smoking cessation.

Unanticipated impacts?

There is an opportunity to develop an evidence base to explore if an integrated approach to a complex set of behavioural changes can work and in what ways, and with what impacts. There is some evidence that attempting to change multiple risk factors at once may be both confusing and difficult to achieve. Provision of information and referral may impact on service providers, particularly in poorer resourced areas where there are limited services for addressing SNAP risk factors, resulting in a higher level of unmet demand for services.

Equity Recommendations for implementation

- *The pilot* to include a strong evaluative component: who uses the service, how similar or different is it from the Quitline, including how it arranges referral to other services, and the impact on other services including unmet demand.
- *Positioning the service* to address access by CALD and isolated rural communities and ensuring the quality of service provided by any subcontracted NGOs for specific groups (eg CALD)
- *Enhance information systems* to develop and maintain links to local services as a referral base

INITIATIVE: COMMUNITY HEALTH CENTRE RISK FACTOR MANAGEMENT

3.1.4 Risk factor assessment and management project

Potential Size of Impact: Medium

Likelihood of Impact: Probable

Populations Affected:

	Whole of Population	Age	Gender	Socio-Economic Position	Ethnicity	Place of Residence
**Identified in Proposal**	✓					
**Added in Appraisal**				✓	✓	✓

What is the initiative trying to do?

The initiative aims to increase the capacity of AHS community health (CH) services to manage chronic disease risk factors in their client populations through healthy lifestyles, and will be informed by findings from current pilot work (CHRFMRP) and the development of CHIME. Within each AHS, CH teams would develop their own locally adapted model of risk factor management (RFM) appropriate to the client group and to service policies and procedures, led by a dedicated position. RFM model development in AHS will undergo ongoing evaluation. Initial reports from CHRFMRP indicate the program is reaching low SES. CHIME has the ability to provide data on postcode, age and ethnicity.

Is there evidence of inequality?

There is little evidence on how well models, for example the 5A's model underpinning CHRFMRP, apply across groups to help change behaviour. An explicit focus on client cultural and linguistic diversity and literacy levels should inform local adaptation of the model.

Who may be disadvantaged by the initiative?

Strategies such as 'goal setting' and 'motivational interviewing' are more suited to those with more resources, capacity and control over their lives, and may advantage the advantaged. It is not clear if all CH Services have the capacity and/or resources to develop systematic programs that will have significant reach into the high-risk populations, particularly in rural areas, so those groups at most risk may have least access to the program. Heavy reliance on written material may not be usable for those groups with low literacy. Provision of SNAP interventions, particularly in clients' homes, needs to be handled sensitively.

Unanticipated impacts?

The flexibility to develop local models may lead to a proliferation of different services that are hard to "scale up" and evaluate at a state level. CH workers have strong competing demands and one dedicated position across each AHS may not be enough to assist all CH centres. A focus on service policies/ procedures may impact on CH centres' ability to provide outreach to groups less likely to access services.

Equity recommendations for implementation

- *Information system* to improve understanding of work currently being done and how this may need to be developed or re-shaped over time to effectively engage in RFM for disadvantaged groups. This will include an understanding of the reach of activities and groups using services.
- *Training and resources* to be assessed for cultural appropriateness and suitable literacy levels.
- *Local service development* to be based on development of general principles and programs to avoid a proliferation of programs that are difficult to evaluate. These principles should include strategies for including disadvantaged groups, including community engagement.
- *Linkage with general practice programs* to optimally use health care resources in disadvantaged communities.
- *Additional item:* This initiative to be explicitly integrated into other chronic disease initiatives (particularly 3.1.2, 3.1.5, 1.3.6, 1.3.5)

PART 4: THE EFHIA PROCESS

4.1 WHAT IS EQUITY FOCUSED HIA?

HIA is a combination of procedures, methods and tools by which a policy, program or project may be assessed and judged for its potential effects on the health of the population and the distribution of these impacts within the population. The NSW HIA and Australasian Collaboration for Health Equity Impact Assessment (ACHEIA) EFHIA projects have identified a set of structured steps to be followed in the HIA process:

1. Screening
2. Scoping
3. Identification and assessment of the potential health impacts[2]
4. Negotiation and decision making
5. Monitoring and evaluation

There are several levels at which a HIA can be undertaken:

- Desk based
- Rapid
- Intermediate
- Comprehensive

Normally a proposal such as the ABHI that has the potential to have a substantial impact on health and where there is substantial resource investment would be subjected to an intermediate or comprehensive HIA. However due to the timeframe within which the assessment needed to be undertaken, it was decided by CHETRE to undertake a Rapid Equity focused HIA using an equity filter.

This part of the report follows the structure of the EFHIA. It details the outcomes of the screening and scoping stages, and describes the processes through which the identification and assessment of health impacts were undertaken, and the process of negotiation and decision-making. Mechanisms are proposed by which the impact of the EFHIA can be monitored and evaluated. The final section includes the reflections of those who participated concerning the strengths and weaknesses of undertaking a rapid EFHIA within a limited timeframe.

[2] The EFHIA framework separates impact identification and assessment of impacts.

4.2 SCREENING

The purpose of screening is to identify the potential links between the policy proposal (in this case ABHI) and health, what links there are to equity and inequalities in health, and whether and in what way the proposal might impact differentially on committees within the population.

The screening report (see below) for the EFHIA was prepared by the CHETRE HIA team prior to the first teleconference. It was based on guidance being developed by the NSW HIA project on HIA in NSW. Based on the report a decision was made by CHETRE to proceed with the HIA and confirmed by the Steering Committee.

Screening Report

This screening report is on the Australian Better Health Initiative Implementation Plan which has been developed as part of a Council of Australian Governments Reform Package aimed at achieving better health for all Australians. A substantial element of the package focuses on a new approach to the prevention, early detection and management of chronic disease.

In NSW Approximately $20 million of new money has been allocated from Treasury for Priority Areas 1 & 3: promoting healthy lifestyles and supporting lifestyle and risk modification.

This Rapid Appraisal has been undertaken to assess the extent to which these strategies adequately consider equity impacts in their implementation. It has been done in a compressed time frame due to the need for the document to be endorsed by the Minister by the end of next week.

This proposal considers strategies in the areas of lifestyle, physical, social and economic environment and the capacity of the health system to impact on these determinants. It is based on the following assumptions:

- Chronic disease is a major and growing health problem in Australia
- It is possible to prevent the risk factors of chronic disease and also to improve people's health and well-being through the adoption of health lifestyles.
- Some parts of the population have poorer health and higher levels of chronic disease than others.
- There is evidence that some strategies have less impact on different population communities, especially vulnerable and "at risk" populations.

This proposal has the potential to positively impact on health by increasing the proportion of the population who lead healthy lifestyles and reducing the number of people with risk factors of chronic disease. The increased level of funding will provide increased resources and incentives for strategies to prevent chronic disease and promote health and well-being. It will also increase the capacity of the health system and other settings (such as schools and child care centres) to effectively deliver these strategies.

There is potential for these strategies to have differential impacts across the population. Many of the strategies many inadvertently exclude populations who are most at risk due to access to services, transport, language and lack of cultural appropriateness. The recently released AIHW Report on Chronic Disease and Associated Risk Factors, 2006 [1] provides detailed information on patterns of health inequality related to socio-economic status and associated risk factors.

In this EFHIA we have chosen to focus on five areas where there are currently health inequalities or potential for health inequalities to develop. These are: age, gender, place of residence, ethnicity and socioeconomic position. The specific needs of Aboriginal people are not considered in this report as this has been done by NSW Health in collaboration with the Aboriginal Health Branch in NSW Health.

The purpose of this rapid EFHIA is to identify ways in which the strategies can be implemented to reduce health inequalities and minimise the potential of the strategies to worsen health inequalities, and to undertake this task within a timeframe that will allow this information to be considered before the plan is finalised.

4.3 SCOPING

The purpose of the scoping step is to set the scope of the EFHIA by establishing the terms of reference for the Steering Committee, clarifying definitions of health and equity, the dimensions of equity to be considered in the EFHIA and planning the process through which the EFHIA will be undertaken and decisions made.

This proposal has a high level of investment of new money and potentially will have significant impacts on the long term health and well-being of the people of NSW. Normally it would warrant an intermediate or comprehensive HIA. However, as the opportunity to influence the proposal was within a very short timeframe (four working days), it was decided to undertake a rapid appraisal using an equity filter. This was in recognition that there may be unintended consequences of the implementation of the proposal that result in increased inequity in accessing and benefiting from preventive programs.

Type of HIA

Rapid Appraisal based on one day workshop and follow up teleconferences to finalise recommendations.

Approach

Equity filter

Parameters of the HIA

In agreeing to have the rapid appraisal undertaken NSW Health set a number of parameters. The details are included in appendix 1. These includes: limiting the number of strategies (8) to be examined and confining the recommendations to existing strategies within existing budgets included in the ABHI proposal.

Steering Committee Members

	**Organisation**	**Expertise**
Elizabeth Harris	CPHCE, UNSW	HIA/ Equity
Patrick Harris	CPHCE, UNSW	HIA
Lynn Kemp	CPHCE, UNSW	Equity / Early Intervention
Nidia Raya Martinez	NSW Health	Health Promotion / Policy analysis
Dave Trudinger	Victorian Department of Human Services	Equity
Michelle Bonner	NSW Council of Social Services	Equity/ Policy analysis
Lesley King	University of Sydney	Health Promotion/ Equity
Also consulted:		
Mark Harris	CPHCE, UNSW	Chronic Disease & Primary Health Care
Rachel Laws	CPHCE, UNSW	Risk Factor Management in Community Settings

Terms of Reference

1. To undertake a rapid appraisal of specific strategies identified by NSW Health using an equity filter.
2. To look specifically at the potential equity impacts on populations identified by age, gender, place of residence, socio-economic position, and ethnicity.
3. To make recommendations on ways in which the implementation of the strategies can be modified to strengthen the likelihood that strategies will reduce health inequality and reduce the likelihood that they will widen health inequalities.
4. To keep the content of the implementation plan and the recommendations of the HIA Rapid Appraisal confidential.

Clarification of values and assumptions

Definition of health

The Committee took a broad definition of health as reflected in the WHO constitution which defines health as a "state of complete physical, social and mental well-being, and not merely the absence of disease or infirmity" Health is seen as a resource for everyday life, not the object of living. It is a positive concept emphasizing social and personal resources as well as physical capabilities.

Definition of equity

"Equity in health implies that ideally everyone should have a fair opportunity to attain their full potential and, more pragmatically, that no one should be disadvantaged from achieving this potential, if it can be avoided. Equity is therefore concerned with creating equal opportunities for health and with bringing health differentials down to the lowest possible level." [2]

Definition of inequity

The term inequity refers to differences in health that are judged to be unnecessary and avoidable but, in addition, are considered to be unfair and unjust.

In this HIA we will therefore consider making recommendations on the implementation of the plan that will:

- improve the overall health of the population, whilst at the same time
- differentially improve the health of those populations who are most vulnerable to reduce the gap between those who are most and least disadvantaged.

4.4 IDENTIFICATION AND ASSESSMENT OF THE POTENTIAL HEALTH IMPACTS

The purpose of 'impact identification' is to collect information to identify the potential and/or actual impacts of the proposal. The purpose of 'assessment of impacts' is to appraise the identified impacts from an equity perspective

Within the timeframe of the HIA it has not been possible to undertake extensive reviews of the literature to identify the impact of specific strategies on the health of the populations identified. We were unable to consult with other key stakeholders, nor with consumer or community committees.

We have relied on a number of key documents and the expertise of the Committee to identify and assess the likely impacts of the proposals.

Key documents consulted

The key documents consulted included:

- AIHW Chronic Disease and Associated Risk Factors in Australia [1]
- AIHW Health Inequality in Australia: Morbidity, health behaviours, risk factors and health service use.[3]
- NSW Chief Health Officers Report
- Australian Better Health Initiative Implementation Plan
- Centre for Chronic Disease Prevention and Health Advancement HIA on the Proposed Integrated Chronic Disease Prevention Social Marketing Campaign [4]
- HIA on Proposed Integrated Chronic Disease Prevention Social Marketing Campaign: Consultant's Literature Review [5]

4.5 PROCESS FOR DEVELOPING RECOMMENDATIONS

The purpose of the 'decision-making and recommendations' stage of EFHIA is to develop a set of solution focussed recommendations for acting on the results of the EFHIA

1. As a committee the Steering Committee held a general discussion on the strategies in the implementation plan and their intended outcomes.
2. Each strategy was reviewed with a specific focus on equity-related issues through the use of the equity filter that asked 5 questions:
    - What is the strategy hoping to achieve?
    - Is there evidence of inequality?
    - Who may be disadvantaged by the initiative
    - Are there likely to be any unanticipated outcomes?
    - What adjustments can be made to make implementation more equitable?
3. The information from the one day workshop was then formatted under each of the strategies with an overall review statement.
4. These drafts were then discussed by the committee in two teleconferences and revised.
5. An interim report was prepared and forwarded to NSW Health. Their comments were considered and a final report was prepared.

4.6 MONITORING AND EVALUATION

The purpose of 'Monitoring and Evaluation' is to systematically consider what added value the EFHIA process brought to the decision making processes in terms of equity considerations.

In May 2007 CHETRE will contact the Area Health Service Directors of Health Promotion and managers within CCDPHA and ask how the EFHIA recommendations have been used to inform implementation of the ABHI plan. Feedback will be provided to the NSW HIA steering committee and the Director of CCDPHA.

4.7 REFLECTIONS ON THE PROCESS

At the end of the first meeting we asked Steering Committee members to reflect on the process that had been undertaken. All members of the committee felt that the process had been worthwhile and would participate in another rapid EFHIA.

The factors that were identified as facilitating the process were:

- the clear parameters of the EFHIA outlined by the Department
- the Screening and Scoping process used existing tools to ensure that key HIA process issues were addressed
- the structured process allowed each strategy in the document to be systematically analysed using the equity filter
- the relatively small size of the committee
- participants from a range of organisations i.e. Health, university and NGO peak body encouraged a range of opinions, expertise and broad discussion
- the strong sense of common purpose
- willingness to accept the work had to be completed within a very tight timeframe
- willingness to make possible and plausible suggestions for implementation within the parameters of the ABHI implementation plan
- willingness of policy makers in NSW health to open their ABHI implementation plan to scrutiny and to consider equity in more detail
- willingness by CHETRE and other participants to scrutinise the plan and initiatives within the realities of the policy context surrounding the plan
- the high level of expertise within the committee across health promotion, equity, policy analysis and chronic disease, and early intervention
- active involvement of policymakers from NSW and Victoria
- clear objectives for the EFHIA enabled recommendations to be structured and worded in a manner relevant to the ABHI implementation plans.
- participation by another state jurisdiction also allowed a wider set of experiences to be drawn upon.

A number of barriers were also identified:

- lack of time to identify and refer to existing evidence bases
- difficulties in participants stepping back from their own strategies to identify potential negative or unanticipated consequences, although participants were explicit about roles in the development in particular strategies, which enabled a balanced discussion to be maintained.

4.8 REFERENCES

1. Australian Institute of Health and Welfare. *Chronic Diseases and Associated Risk Factors in Australia, 2006, Cat. No PHE 81* Canberra: AIHW, 2006.
2. Whitehead M, editor. *The Concepts and Principles of Equity in Health*. Copenhagen: World Health Organisation Regional Office for Europe, 1990.
3. Turrell G SL, de Looper M & Oldenburg B. *Health Inequalities in Australia: Morbidity, health behaviours, risk factors and health service use. Health Inequalities Monitoring Series No. 2. AIHW Cat. No. PHE 72.* . Canberra: : Queensland University of Technology and the Australian Institute of Health and Welfare., 2006.
4. Centre for Chronic Disease Prevention and Health Advancement. Health Impact Statement (Final Report). Sydney: Centre for Chronic Disease Prevention and Health Advancement, NSW Department of Health, 2004.
5. Eureka Strategic Research. Literature Review for the Centre for Chronic Disease Prevention and Health Advancement HIA on the Proposed Integrated Chronic Disease Prevention Social Marketing Campaign, 2004.

APPENDIX 1: COMMISSIONING BRIEF

Project

Equity Filter applied to Australian Better Health Initiative (ABHI) implementation plan.

Background

A consultation paper has been developed for the components of the ABHI for which the Centre for Chronic Disease Prevention and Health Advancement are responsible. This paper details a range of initiatives in response to priorities 1 and 3 of the ABHI and has been developed in consultation with a wide range of key stakeholders, including:

- Other divisions within NSW Health Department – Aboriginal Health Branch, Inter Government Funding & Strategies Branch and Chronic Care Program
- Directors of Health Promotion - Area Health Services
- Directors of Population Health & Planning – Area Health Services
- Research Organisations (including Centre for Physical Activity & Nutrition, Centre for Overweight & Obesity and Centre for Public Health Nutrition)
- Collaborative Centre for Aboriginal Health Promotion
- Centre for Health Equity Training Research & Evaluation
- School of Community Medicine, UNSW
- Non Government Organisations (including the National Heart Foundation, Diabetes Australia and Cancer Council NSW)
- Chair of the NSW Population Health Priority Taskforce

During a feedback session held with the Centre for Health Equity Training Research and Evaluation on November 15, it was suggested that latest draft of the implementation plan would benefit from an equity filter/audit. The purpose of the equity filter would be to:

1. make practical recommendations in order to improve the equity of proposed strategies and
2. ensure that the strategies do not unintentionally widen the equity gap.

Discussions within the Centre for Chronic Disease Prevention and Health Advancement gave tentative support to this suggestion, however the following concerns were raised:

- Timeframe – the implementation plan needs to be submitted through the Department to the Minister's office by November 24, hence advice must be provided by 22 November.
- There is a need for specific recommendations that improve the equity of existing strategies within existing budget parameters;
- Not being able to fulfil all expectations due to the competing interests.
- Some of the initiatives are primarily focused on building the intervention effectiveness evidence base and as a result with have less of an equity focus.

Accordingly, the Centre supports the progression of the equity filter project within the following parameters.

Parameters of the Project

Purpose of project

To apply an equity filter to proposed ABHI initiatives and provide practical recommendations on improving the reach of these initiatives in disadvantaged communities.

Process of the project

Rapid equity filter audit undertaken by members of CHETRE.

Focus of the equity filter

The project should focus on these areas of disadvantage:

- Aboriginal and Torres Strait Islander communities
- Relevant culturally and linguistically diverse communities
- Locational disadvantage (rural and remote communities).

Other priority populations as considered appropriate.

Timeframe

Recommendations are required by COB on Wednesday 22 November to allow sufficient time for the Centre to seek Ministerial approval by Friday 24 November.

Initiatives to be audited include

Promoting Health Lifestyles

1.3.1 Community based healthy weight program – Aboriginal and Torres Strait Islander communities

1.3.2 Chronic disease prevention funding program – local government and urban planning setting

1.3.3 Chronic disease prevention funding program – child care setting

1.3.4 Chronic disease prevention funding program – school setting

1.3.5 Chronic disease prevention funding program – community setting

1.3.6 Physical activity provider capacity building project

Supporting Lifestyle and Risk Modification

3.1.1 Community based diabetes prevention program - pilot

3.1.2 Lifestyle and risk factor modification information service

3.1.4 Risk factor assessment and management project

Issues outside the scope of the project

The project is to provide recommendations and advice on the implementation of the above-mentioned strategies; it is outside the scope of the project to propose new strategies.

CHETRE is to be mindful in the drafting of recommendations, that the primary focus of some of the proposed strategies (eg: community based diabetes prevention program and lifestyle information service) is the building of the intervention feasibility and effectiveness evidence base.
